# Readability of Online Materials for Rhinoplasty

**Published:** 2018-01

**Authors:** Pauline Joy F Santos, David A Daar, Keyianoosh Z Paydar, Garrett A Wirth

**Affiliations:** Department of Plastic Surgery, University of California, Irvine, USA

**Keywords:** Readability, Rhinoplasty, Patient, Education

## Abstract

**BACKGROUND:**

Rhinoplasty is a popular aesthetic and reconstructive surgical procedure. However, little is known about the content and readability of online materials for patient education. The recommended grade level for educational materials is 7th to 8th grade according to the National Institutes of Health (NIH). This study aims to assess the readability of online patient resources for rhinoplasty.

**METHODS:**

The largest public search engine, Google, was queried using the term “rhinoplasty” on February 26, 2016. Location filters were disabled and sponsored results excluded to avoid any inadvertent search bias. The 10 most popular websites were identified and all relevant, patient-directed information within one click from the original site was downloaded and saved as plain text. Readability was analyzed using five established analyses (Readability-score.com, Added Bytes, Ltd., Sussex, UK).

**RESULTS:**

Analysis of ten websites demonstrates an average grade level of at least 12^th^ grade. No material was at the recommended 7^th^ to 8^th^ grade reading level (Flesch-Kincaid, 11.1; Gunning-Fog, 14.1; Coleman-Liau, 14.5; SMOG 10.4; Automated Readability, 10.7; Average Grade Level, 12.2). Overall Flesch-Kincaid Reading Ease Index was 43.5, which is rated as “difficult.”

**CONCLUSION:**

Online materials available for rhinoplasty exceed NIH-recommended reading levels, which may prevent appropriate decision-making in patients considering these types of surgery. Outcomes of this study identify that Plastic Surgeons should be cognizant of available online patient materials and make efforts to develop and provide more appropriate materials. Readability results can also contribute to marketing strategy and attracting a more widespread interest in the procedure.

## INTRODUCTION

The use of the Internet has increased across all ages and demographics in the United States. Over the past ten years, Internet use in the US has risen from 52% to 85%, and a recent consumer survey showed that search engines in particular are used to identify sources of information.^1^^,^^2^ Additionally, the use of the Internet has become an important resource for health information. According to the Health Information National Trends Survey, 48.6% of persons go online for health information before visiting a physician.^3^ Furthermore, patients use the Internet to further investigate information provided by their healthcare professional.^4^

Patient education is a critical component of shared decision-making, which has increased patient satisfaction and improved health outcomes.^5^^,^^6^ With the increasing use of the Internet, healthcare professionals should be aware of appropriate online patient education materials. When developing and evaluating such materials, it is critical to consider health literacy. Health literacy is defined as the patient’s ability to understand basic health information and services to make an appropriate healthcare decision.^7^ By providing appropriate educational materials, healthcare providers can combat the sequelae of lower health literacy, which include negative impacts on health, barriers to receiving care, and increased mortality.^8^^-^^10^

Previous recommendations by the American Medical Association (AMA) suggested that patient education materials should be at the 6th grade level.^11^ The National Institutes of Health (NIH) released an updated recommendation that patient education materials should be at the 7th or 8th grade level.^12^ However, several studies evaluating the readability of patient education material of a variety of healthcare topics have revealed an average grade level of at least 12th grade.^13^^-^^20^ In a study assessing the readability of patient education materials from surgical subspecialties, the readability ranged from 10th- to 15th-grade level.^21^

Patient-directed documents on the American Society of Plastic Surgeons (ASPS) and American Society for Aesthetic Plastic Surgery (ASAPS) websites were consistently rated as difficult to read when compared to other health information websites.^22^ In another study evaluating patient health education material found on the American Academy of Facial Plastic and Reconstructive Surgery (AAFPRS) website, the articles were found to be written at an average grade level of 12th grade using 10 different readability scales. The articles on rhinoplasty were at least at the 10^th^ grade level.^13^

Rhinoplasty is the third most common aesthetic surgical procedure performed.^23^ The role of the Internet in providing information for rhinoplasty is increasingly important. In a survey study of patients undergoing post-traumatic or aesthetic rhinoplasty, patients searched online for description of operations, contact with other patients and with surgeons, and for preoperative and postoperative pictures. The study suggests that surgeons should provide appropriate websites to improve patient-physician communication.^24^^,^^25^ The primary aim of this study was to evaluate the readability of available Internet resources for patient information about rhinoplasty through the use of well-established instruments for assessing readability of educational content.

## MATERIALS AND METHODS

A Web search for “rhinoplasty” was performed using Google, and the top 10 sites were identified. Location, cookies, and user account information were disabled before each search to avoid inadvertent bias in the results returned. Sponsored hits were excluded. The included sites in order of visit frequency were the following: Plasticsurgery.org, Wikipedia.org, Realself.com, Nlm.nih, Aafprs.org, Webmd.com, Rhinoplasty.com, Mayoclinic.com, Rhinoplastysociety.org, and Newyorkfacialplasticsurgery.com (Table 1). All sites were accessed on February 26, 2016. Patient-directed content from all relevant articles that were greater than 100 words and directly accessible from the original parent site was downloaded. Content was formatted into plain text in separate Microsoft Word 2011 documents (Microsoft Corp., Redmond, WA). 

**Table 1:** Websites accessed

**Website **	**Organization**	**Number of articles**
Plasticsurgery.org	American Society of Plastic Surgeons	9
Wikipedia.org	Wikipedia	8
Realself.com	RealSelf, Inc.	8
Nlm.nih	Medline Plus/National Library of Medicine	1
Aafprs.org	American Academy of Facial Plastic and Reconstructive Surgery	1
Webmd.com	WebMD	2
Rhinoplasty.com	Geoffrey Tobias, MD	7
Mayoclinic.com	Mayo Clinic	2
Rhinoplastysociety.org	The Rhinoplasty Society	5
Newyorkfacialplasticsurgery.com	New York Center for Facial Plastic & Laser Surgery	1
Total		44

A total of 44 articles were downloaded and organized by website. Each article was then edited to exclude images, videos, figures, captions, advertisements, references, links, disclaimers, and acknowledgements. Forty-four articles were analyzed using the readability software. Readability assessment was performed using Readability-Score.com. First, all 44 rhinoplasty articles were analyzed together; subsequent analysis was performed on each group of articles arranged by parent website for comparison. The readability of each group was assessed using 6 established tests: Flesch-Kincaid Reading Ease, Flesch-Kincaid Grade Level, Gunning Fog Score, Coleman Liau Index, SMOG Index, and Automated Readability Index (Table 2).^26^

**Table 2:** Tests for readability analysis

**Test**	**Score Type**	**Qualities Assessed**	**Formula**
Flesch-Kincaid Reading Ease	Index score range (0-100, where 100=easiest)	Word complexity, sentence length	206.835 – (1.015 x ASL) – (84.6 x ASW)
Flesch-Kincaid	Grade Level	Word complexity, sentence length	(0.39 x ASL) + (11.8 x ASW) - 15.59
Gunning Fog Score	Grade Level	Word complexity, sentence length	0.4 (ASL + PHW)
Coleman Liau Index	Grade Level	Word complexity, sentence length	0.0588L – 0.296S – 15.8
SMOG Index	Grade Level	Word complexity, sentence length	3 + Square Root of Polysyllable Count
Automated Readability Index	Grade Level	Word complexity, sentence length	4.71 (characters/words) + 0.5 (words/sentences) – 21.43

ASL: Average sentence length (i.e., the number of words divided by the number of sentences); ASW: Average number of syllables per word (i.e., the number of syllables divided by the number of words); PHW: Percentage of hard words; L: The average number of letters per 100 words; S: The average number of sentences per 100 words

## RESULTS

**Fig. 1:**
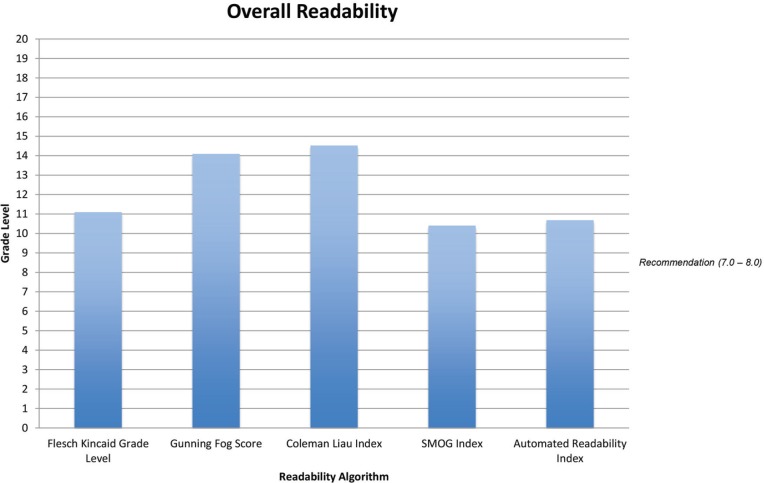
The overall readability for all ten websites exceeded the NIH-recommended 7^th^ to 8^th^ grade level according to analysis using 5 different readability algorithms. For each readability algorithm, the grade level was at least 10^th^ grade.

Health information available from the 10 most frequently used websites about rhinoplasty had an overall average grade level of 12.2. The overall readability (by grade level) of all websites was determined by 5 algorithms (Figure 1). The mean Flesch-Kincaid grade level was 11.1*.* The mean Gunning-Fog grade level was 14.1, with a range from 9.7 to 20.9. The mean Coleman-Liau grade level was 14.5, with a range from 11.3 to 18.2. The mean SMOG grade level was 10.4. The mean Automated Readability grade level was 10.7. The Flesch-Kincaid Reading Ease evaluation provides an index score from 0 to 100, with 100 being easiest to read. The mean Flesch-Kincaid Reading Ease Index was 43.5, which is rated as “difficult.” 

**Fig. 2:**
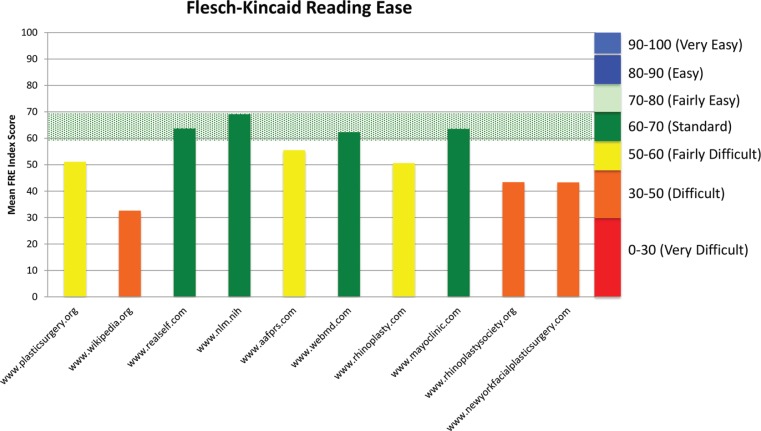
The reading ease of three websites was rated as “difficult,” which is a score of 30-50. Three were rated as “fairly difficult,” which is a score of 50 to 60, and four websites were rated as “standard,” which is a score of 60-70. No website was rated as “fairly easy,” which is a score of 70 to 80.

**Fig. 3:**
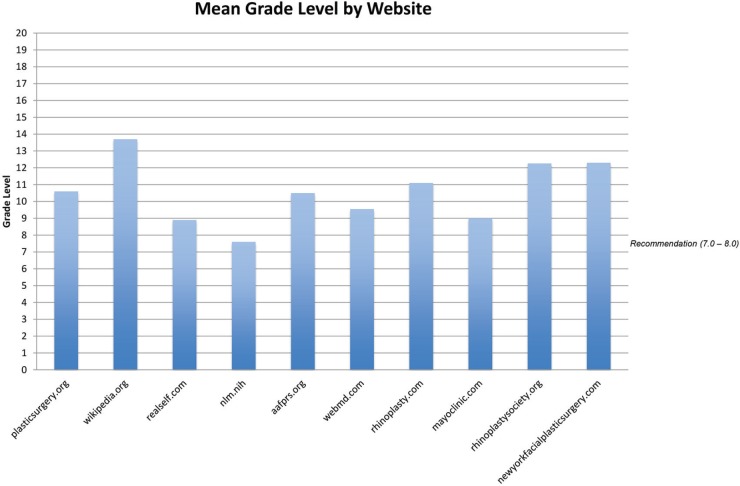
The mean grade level by website was above the NIH-recommended 7^th^ to 8^th^ grade level for all websites with the exception of the Nlm.nih website.

**Fig. 4:**
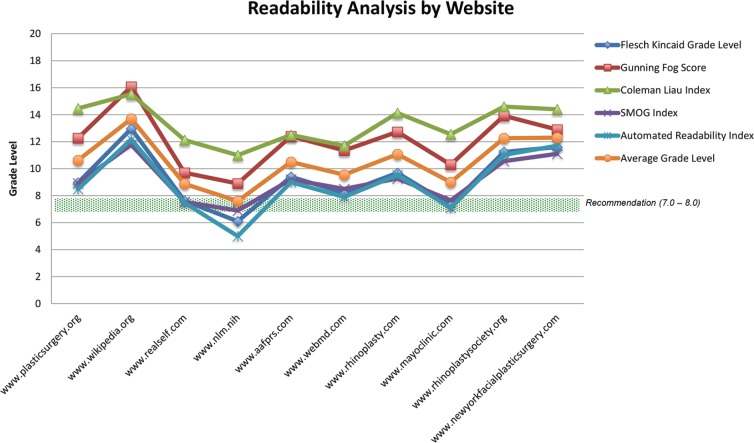
The grade level by most readability algorithms was above the recommended reading level, and the average grade level was above the recommended reading level for all websites.

Analysis by parent site revealed a spectrum of readability, both by Flesch-Kincaid Reading Ease (Figure 2) and by average grade level (Figures 3 and 4). Flesch-Kincaid Reading Ease index score ranged from 69 (Nlm.nih.gov) to 33 (Wikipedia.org). Of note, a score of 69 is rated as “standard,” while a score of 33 is rated as “difficult.” Site difficulty was consistent with analysis by overall average reading level, which ranged from 7.6 (Nlm.nih.gov) to 14 (Wikipedia.org). 

## DISCUSSION

The Internet has greatly increased access to patient education resources regarding elective cosmetic as well as reconstructive procedures. However, several recent studies have demonstrated that the readability of online patient materials in plastic surgery exceeds the NIH-recommended reading level of 7^th^ to 8^th^ grade.^14^^-^^20^^,^^27^ This may be particularly critical with regard to rhinoplasty, considering its popularity as an aesthetic surgical procedure.^23^ To our knowledge, our study is the first to assess the reading level of Google search engine query results for rhinoplasty patient education materials. 

Of the top 10 websites evaluated in this study, only the NIH website (Nlm.nih.gov) provided patient material at the recommended reading level. In fact, the average grade level of the 44 articles analyzed was above a 12^th^ grade reading level. In a previous readability study, the readability of health information from popular websites was compared with the readability of popular magazines including National Geographic, People Magazine, Reader’s Digest, Sports Illustrated, and Time Magazine. The average grade level of popular magazines was 9.5.^16^

This comparison demonstrates that grade levels determined by readability algorithms are reliable measures of content complexity. In an article describing the design of websites for rhinoplasty and facial plastic surgery, Becker agrees that the Internet is a critical component of developing one’s plastic surgery practice and recommends providing appropriate health information. He comments that his patients are “quite sophisticated in their level of understanding.” Similarly, a study evaluating the comprehension of breast augmentation and rhinoplasty presented by the ASPS and ASAPS websites among 100 patients concluded that participants understood the majority of information presented to them. However, this study is limited in that its patient population had a mean number of completed educational years of 11.7.^28^

While these studies imply a sufficient understanding of patient education materials on rhinoplasty, their samples are limited to “sophisticated” and highly educated patients. If health information on rhinoplasty is presented at a grade level more representative of national literacy and according to recommended reading levels, the scope of patients interested in the procedure may broaden. Rhinoplasty is a common cosmetic and reconstructive procedure in the teenage population. According to the ASPS, in 2013, there were 30,672 patients age 13-19 years who underwent rhinoplasty, accounting for almost 50 percent of all cosmetic procedures among teenagers.^29^

While both patient and parent consent are crucial and mandatory for teens under age 18, consent must be paired with sufficient understanding of the procedure itself. If online reading materials are at an average reading level of a 12^th^-grade student, a significant portion of these teenagers may not have even had the reading training necessary to comprehend the information conveyed. Due to the complexity of the rhinoplasty procedure and the incredibly wide array of surgical techniques, patients considering rhinoplasty benefit from guidance.^25^

Thus, it is imperative that plastic surgeons distill the procedural information down into media that does not overwhelm them. Previous studies have suggested providing patients with as much informational material as possible.^30^ However, the results of this study potentially suggest that rather than a deluge of online information, the appropriateness and concision of materials provided may be more effective. In addition, while this study did not include images, videos, diagrams, etc., as a means of conveying information to patients, these methods may offer synergy with text in visually presenting the various treatment options. More research is warranted in identifying the use of multimedia information for rhinoplasty patients.

Appropriate patient education material not only affects patients considering rhinoplasty-this study provides insight into online marketing for plastic surgeons. A 2013 study by online ad network, Chitika, showed that page one in US and Canadian Google search results receives 91.5% of all traffic.^31^ Therefore, this study has implications for the top websites we identified as well as individual aesthetic surgery practices that may be filtered to the patient’s front page based on promotion or location. While it is necessary for the top 10 unfiltered websites to improve the readability of their materials, it is the duty of locally-based surgeons to ensure their websites meet the literacy needs of their patients. 

As the majority of surgeons perform some form of online marketing,^32^ these search engine filters offer an opportunity to deliver effective direct marketing materials. It makes sense, then, that this information be at the recommended reading level for their potential patients. In contrast to previous studies on cosmetic procedures, e.g., breast augmentation,^14^ our study of online rhinoplasty materials delivered Realself.com as a top 10 result. As a popular social media forum and patient-rating site, the readability level of Realself.com relies on both the written information surgeons provide in direct response to patient questions as well as patient reviews.^33^^,^^34^

Notwithstanding the accuracy of content, this poses a major challenge for surgeons performing rhinoplasty, as they must consider the appropriate literacy level for each response they deliver. As social media continues to transform the Internet landscape, surgeons have the ability to engage in an ongoing dialogue with their current and future patients.^32^ By proactively delivering accurate, literacy-appropriate information on rhinoplasty, plastic surgeons can attenuate patient dissatisfaction due to poor understanding. This is particularly important in rhinoplasty, as patient dissatisfaction remains relatively high in both male and female patients.^34^^,^^35^

One study illustrated that rhinoplasty does not have an effect on general health nor on quality of life, but rather an improvement in psychological health.^36^ As such, identifying patient desires and assessing patient understanding is vital to achieving patient satisfaction and enhancing the patient-physician relationship.^35^^,^^37^ Constantidinides describes the rhinoplasty consultation and business of rhinoplasty being dependent on the patient-physician relationship. He says “Establishing a relationship with our patients is crucial before agreeing to surgery.” He then describes a situation in which he believed he achieved objectively excellent results but his patient “saw only problems”.^38^

A recent study of rhinoplasty patients demonstrated that satisfaction was the most important measure of successful surgical outcomes and was highly dependent on the patient’s perception about treatment.^39^ Part of creating a successful patient-physician relationship includes appropriate information sharing and optimizing shared-decision making.^40^ A key aspect of the decision making process is creating reasonable patient expectations, which can be enhanced by providing the appropriate educational materials before surgical intervention. While previous readability studies have assessed content using sophisticated and relatively expensive tools for analysis, we purposely utilized free software found at Readability-score.com. While our results may not be as complex and granular, the NIH cites Readability-score.com as an effective tool for evaluating the grade level of patient health information.^12^

The ability to easily access readability software can expedite improvement of current knowledge of online patient materials, and its use before publication of future health information can be an important step to match the health literacy of patients. The Internet has provided exceedingly easy access to health information, without our judgment as to reliability of that information. Plastic surgeons should take responsibility for directing their patients to appropriate websites that provide correct information and developing patient education material that is easy to understand. This study demonstrates that the most frequently visited websites for rhinoplasty information currently exceed recommended health-literacy recommendations and require content revision for optimal patient-physician communication and health outcomes in order to align with recommendations such as those from the NIH. 
